# Diagnostic Accuracy of the Drug Use Disorder Identification Test and Its Short Form, the DUDIT-C, in German Adolescent Psychiatric Patients

**DOI:** 10.3389/fpsyg.2021.678819

**Published:** 2021-06-04

**Authors:** Lukas A. Basedow, Sören Kuitunen-Paul, Anna Eichler, Veit Roessner, Yulia Golub

**Affiliations:** ^1^Department of Child and Adolescent Psychiatry, Faculty of Medicine, Technische Universität Dresden, Dresden, Germany; ^2^Department of Child and Adolescent Mental Health, University Hospital Erlangen, Friedrich-Alexander University Erlangen-Nürnberg, Erlangen, Germany

**Keywords:** psychoactive drugs, DUDIT, adolescence, cut-off, ROC curve, substance use disorder, screening

## Abstract

**Background:**

A common screening instrument for substance use disorders (SUDs) is the Drug Use Disorders Identification Test (DUDIT) which includes a short form regarding only drug consumption (DUDIT-C). We aim to assess if a German version of the DUDIT, adapted for adolescents, is a suitable screening instrument in a sample of adolescent psychiatric patients.

**Methods:**

*N* = 124 (54 female) German adolescent (*M* = 15.6 + 1.5 years) psychiatric patients completed the DUDIT and received a diagnostic interview (MINI-KID) assessing DSM-5 SUD criteria. A confirmatory factor analysis (CFA), receiver operating characteristic (ROC) curves, the area under the curve (AUC), and Youden’s Index were calculated.

**Results:**

A two-factor model of the DUDIT shows the best model fit (CFI = 0.995, SRMR = 0.055, RMSEA = 0.059, WRMR = 0.603). The DUDIT as well as the DUDIT-C show high diagnostic accuracy, with AUC = 0.95 and AUC = 0.88, respectively. For the DUDIT a cut-off value of 8.5 was optimal (sensitivity = 0.93, specificity = 0.91, *J* = 0.84), while for the DUDIT-C the optimal cut-off value was at 1.5 (sensitivity = 0.86, specificity = 0.84, *J* = 0.70).

**Conclusion:**

This is the first psychometric evaluation of the DUDIT in German, adolescent psychiatric outpatients, using the DSM-5 diagnostic criteria. The DUDIT as well as the DUDIT-C are well suited for use in this population. Since in our sample only few patients presented with a mild or moderate SUD, our results need to be replicated in a sample of adolescents with mild SUD.

## Introduction

About 1 in 20 (4.3%) German adolescents and young adults are diagnosed with a substance use disorder (SUD) related to illicit substances at some point in their life (Perkonigg et al., 2006). A SUD diagnosis in adolescence is associated with a number of additional impairments such as lower school performance, higher mortality rates, and worse overall health (Rattermann, 2014; Schulte and Hser, 2014; Lindblad et al., 2016).

However, adolescents are often not assigned to appropriate treatment plans because SUD symptoms are not identified (Sterling et al., 2010). An accurate and quick recognition of a SUD in adolescents is particularly important since faster treatment initiation is associated with higher engagement and success rates (Chi et al., 2006). The adaptation of SUD criteria of the Diagnostic and Statistical Manual of Mental Disorders-5 (DSM-5) was meant to serve a purpose of precise and fast identification (Hasin et al., 2013). However, extensive verbal screening, a method applied by most psychiatric care givers, lacks in diagnostic accuracy because of the little time available to most practitioners, a need for intricate knowledge of diagnostic criteria, and adolescents being unwilling to provided sensitive information in direct personal contact (Palmer et al., 2019). In addition, adolescents might be lacking insight into the severity of their substance use problems (Wu et al., 2011), i.e., not reporting their drug use because they assume that these are transient issues (Kuznetsova et al., 2016).

To ensure fast identification of adolescent SUDs, care providers need an easy-to-apply, widely available screening tool with high diagnostic accuracy. One such instrument, available in 23 languages and for free from the European Monitoring Centre for Drugs and Drug Addiction (EMCDDA), is the Drug Use Disorders Identification Test (DUDIT). The DUDIT was developed in 2005 based on data from Swedish adults in the criminal justice system, addiction treatment centers and community samples (Berman et al., 2005). The DUDIT was specifically developed as a screening instrument with optimal simplicity, that helps health care professionals to gain a quick impression of problematic illegal drug use (therefore excluding alcohol and tobacco use) in adults (Berman et al., 2005). A review of 18 studies has shown that the DUDIT is a reliable and valid instrument for clinical use, with high internal consistency, sensitivity, and specificity (Hildebrand, 2015). However, only three international studies used the DUDIT within adolescent samples from Turkey (Evren et al., 2014b), South Africa (Martin et al., 2014), and Netherlands (Hillege et al., 2010); evaluating merely internal consistency and factor structure and thus making no judgment about applicability for clinical use. Consequently, DUDIT cut-off values for a SUD screening are available only for adults and are based on the criteria of the Diagnostic and Statistical Manual of Mental Disorders-IV (DSM-IV) of substance abuse and substance dependence (American Psychiatric Association, 2000). According to these DSM-IV criteria, a DUDIT score >24 indicates dependence, independently of gender, while for men a DUDIT score >5 and women >1 indicates a large deviation from the mean in a general population sample (Berman et al., 2005). In addition to using the DUDIT questionnaire, it is possible in terms of a short version to analyze only the first four questions focused on drug consumption (DUDIT-C). In the similar Alcohol Use Disorders Identification Test (AUDIT) the use of a short version (AUDIT-C) as a simplified screening instrument for adolescents is well established (Kuitunen-Paul et al., 2018; Liskola et al., 2018). While the DUDIT-C has been utilized in several studies (Hillege et al., 2010; Willem et al., 2011; Sinadinovic et al., 2012, 2014a,b; Berman et al., 2015; Gidhagen et al., 2017; Bright et al., 2018), the diagnostic accuracy has not been evaluated in adolescents or adults, and therefore no appropriate cut-off values have been published.

To achieve the goal of a high diagnostic accuracy, the cut-off values of an instrument need to allow categorizing a patient correctly as having or not having a disorder (true positives and true negatives). At the same time the cut-off value should minimize the chance of categorizing someone with a disorder as disorder free or vice versa (false negatives and false positives). A common tool to determine appropriate cut-off values is the Receiver Operating Characteristics (ROCs) curve that plots sensitivity (true positive rate) against the false positive rate, which is calculated with 1 – specificity (the true negative rate). Using the ROC curve the area under the curve (AUC) serves as a global measure of discriminative power, while an index that maximizes sensitivity and specificity (Youden’s Index) can be calculated to find a balanced cut-off point for a dichotomous diagnostic test (Fluss et al., 2005) such as the DUDIT.

In our study, we evaluated if the DUDIT can distinguish German adolescent, psychiatric patients with a SUD related to illicit drugs, from adolescent, psychiatric patients without a SUD. To determine if the separation of the questionnaire into a DUDIT-total and DUDIT-C score, as previously reported for the AUDIT (Liskola et al., 2018), is meaningful we performed a confirmatory factor analysis (CFA). Additionally, we aimed to assess discriminant validity (the degree to which different questionnaires measure different concepts) of the DUDIT by comparing it with a questionnaire assessing life satisfaction. By assessing the ROC curve, evaluating the AUC, and calculating cut-off values based on Youden’s Index, we assessed the suitability of the DUDIT-total and DUDIT-C as screening instruments for a SUD related to illicit substances, among adolescent psychiatric patients. Cut-offs indicate the presence of a SUD of any severity according to DSM-5 criteria (American Psychiatric Association, 2013) with a balanced combination of sensitivity (% of true positive subjects within the SUD group) and specificity (% of true negative within the no-SUD group).

## Methods

### Participants

We recruited participants from the general outpatient department and the outpatient department for adolescent substance abuse of our Clinic of Child and Adolescent Psychiatry, Faculty of Medicine, TU Dresden, Germany. This mixed sample was then divided into two groups based on their DSM-5 SUD diagnosis (presenting with at least 2 out of 11 criteria) established with the Mini-International Neuropsychiatric Interview for Children and Adolescents (MINI-KID): Adolescent patients with SUD [*n* = 57 (20 female), mean age = 15.8 ± 1.4 years] and adolescent patients without a SUD [*n* = 67 (34 female), mean age = 15.4 ± 1.6 years].

### Procedure

Between January 2019 and February 2021, we recruited adolescent patients, age 11–18 years, for participation in the study. Patients were approached for recruitment during their first appointment at our clinic. During this first appointment, next to standard clinical procedure LAB, SK-P or a fellow psychologist from our clinic provided an overview over the study and asked for informed consent. *N* = 294 patients were asked to participate in this manner, of which *n* = 249 provided informed consent, meaning *n* = 45 declined to participate. If participants provided consent, we performed the data collection in the form of questionnaires and interviews with patients. The questionnaires were handed out during the first appointment to the participants without further instruction and the request to fill them out alone. The MINI-KID was conducted by a professional psychologist during a separate appointment at our clinic. We only included participants who had fulfilled out the DUDIT as well as participated in the second appointment for MINI-KID, leaving us with *n* = 128 participants (*n* = 48 from the general outpatient clinic and *n* = 80 from the department of substance abuse). The study was conducted in accordance with the Declaration of Helsinki. Patients as well as legal guardians were informed about the projects thoroughly and comprehensively. Written informed consent was obtained from all legal guardians. All procedures of this study were approved by the Institutional Review Board of the University Hospital C. G. Carus Dresden (EK 66022018).

### Measures

The Drug Use Disorders Identification Test (Berman et al., 2005), German version from EMCDDA (2020) is a self-report instrument composed of 11 items identifying problems related to the use of illegal drugs. Scoring of the DUDIT is twofold: items 1 to 9 are scored on a five-point Likert scale, while items 10 and 11 are scored on a three-point scale (with the three items being scored 0, 2, and 4, respectively). The overall score (DUDIT-total) is calculated by summing the scores on all items, with a maximum score of 44. To receive the DUDIT-C score, the scores of the first four questions are summed up, with a maximum score of 16. Previous research in adults established a score of >24 for both sexes as a cut-off score for dependence (Berman et al., 2005). A group of senior psychotherapists and psychiatrist from our clinic adapted the language of the DUDIT to be more appropriate for the adolescent participants (e.g., changing the German formal version of you “Sie” to the more familiar form “Du”). *N* = 85 of our participants were asked to rate the quality and comprehensiveness of the adapted DUDIT questionnaire. The majority (66%) rated the DUDIT as a moderate to good questionnaire (a score of 5 or above on a 10-point scale), about 86% reported that the DUDIT is comprehensible (a score of 5 or above on a 10-point scale), and 96% answered that they understood the majority of the questions in the DUDIT. Consequently, it can be assumed that the DUDIT is well understood by and applicable for adolescents.

The Mini-International Neuropsychiatric Interview for Children and Adolescents (Sheehan et al., 2010) is a structured diagnostic interview used to evaluate the presence of a psychiatric disorder in children and adolescents. The interview uses screening and diagnostic yes/no questions to assess the presence of 32 psychiatric disorders according to DSM-5 criteria. All interviews were conducted by psychologists working in the Department of Adolescent Substance Abuse using a German translation of the original MINI-KID (Plattner et al., 2012). Since the DUDIT only refers to illegal drugs, the main outcome of interest was the presence and severity (mild, moderate, and severe) of a SUD (except alcohol or tobacco) according to DSM-5 criteria.

The Satisfaction With Life Scale (SWLS) Diener et al. (1985), German version from (Glaesmer et al., 2011), is a short self-report instrument on which participants indicate their agreement to five statements about life satisfaction on a seven-point Likert scale. A maximum score of 35 can be reached, indicating a high level of life satisfaction.

### Statistical Analysis

We conducted the CFA with the lavaan package (Rosseel, 2012) in RStudio (RStudio Team, 2020). All other analyses were conducted with IBM SPSS Statistics 25.0. In case of missing values on the DUDIT, participants were excluded if they answered less than 80% of the questions. In cases were at least 80% of questions were answered, missing values were replaced by the mean value of the answered items (*n* = 10). Of *n* = 128 participants, *n* = 4 participants answered less than 80% of the questions, leaving us with *n* = 124 participants for our analyses. Participants were divided into two groups according to their SUD status for the past 12 months (any SUD vs. no SUD) based on the MINI-KID results. Descriptive group differences were *t*- or Chi-Square-tested.

The adequacy of the one-factor model (all DUDIT items), the two-factor model (factor1 = DUDIT-C and factor 2 = DUDIT items 5–11), and the complex model that assumes two meaningful sub-scores that can be combined into a total score, were tested with CFA, using the diagonally weighted least-squares (DWLS) method of estimation to account for non-normality within the categorical items. A good absolute model fit would be indicated by a Chi-square to degrees of freedom ratio smaller than 2 (a ration between 2 and 3 is acceptable), a comparative fit index (CFI) above 0.95 (0.90–0.94 acceptable), a standardized root mean square residual (SRMR) below 0.05 (0.05–0.10 acceptable), and a root mean square error of approximation (RMSEA) below 0.05 (0.05–0.10 acceptable) (Schermelleh-Engel et al., 2003), as well as a weighted root mean square residual (WRMR) below 0.90 (0.90–1.0 acceptable) (DiStefano et al., 2018). The best model can be selected through comparison of the CFI values, with higher values indicating a better fitting model.

In the next step, we aimed to assess discriminant validity. Therefore, we added all SWLS items loading on a single SWLS factor to the CFA model with the best fit. Discriminant validity was accepted if the fit indices were acceptable and similar to the indices from the base model.

In the next step, we created ROC curves to examine sensitivity and specificity at each possible cut-off value by plotting sensitivity (in %) at the *y*-axis vs. 100 – specificity (in %) at the *x*-axis. We calculated the AUC (0–1 range, higher scores indicating higher discriminative power) to assess the overall diagnostic accuracy of the DUDIT. An AUC of 0.7–0.8 is considered as acceptable, 0.8–0.9 is considered to be excellent, and a value higher than 0.9 is outstanding (Mandrekar, 2010). The optimal cut-off point was determined by Youden’s Index *J* (Sensitivity + Specificity − 1; 0–1 range, higher scores indicating higher effectiveness). This procedure was followed for the assessment of DUDIT and DUDIT-C scores. Additionally, we repeated the analysis described above comparing only the participants with a mild or moderate SUD (*n* = 21) to the participants without SUD. A *p*-value < 0.05 was considered significant.

## Results

### Descriptive Statistics

The demographic and clinical characteristics of the sample are presented in Table 1. SUD and non-SUD patients did not differ significantly in mean age or sex. DUDIT-total [*t-score* (122) = −13.3, *p* < 0.001] and DUDIT-C [*t-score* (122) = −9.3, *p* < .001] scores were significantly higher in the SUD group than in the non-SUD group.

**TABLE 1:** Sample description.

	Non-SUD patients (*n* = 67)	SUD patients (*n* = 57)	Test statistic	*p*-Value	Total (*n* = 124)
Mean age in years (SD)	15.4 (1.6)	15.8 (1.4)	*t* (122) = −1.4	0.17	15.6 (1.5)
Sex	34 f, 33 m	20 f, 37 m	*X*^2^ (1) = 3.1	0.08	54 f, 70 m
Mean DUDIT-total score (SD)	2.0 (4.8)	17.7 (8.1)	*t* (122) = −13.3	<0.001	9.2 (10.2)
Mean DUDIT-C score (SD)	0.9 (2.3)	6.1 (3.7)	*t* (122) = −9.3	<0.001	3.3 (4.0)
SUD diagnoses			*X*^2^ (3) = 124.0	<0.001	
No SUD	67	0			67
Mild	0	9			9
Moderate	0	12			12
Severe	0	36			36

*SUD diagnoses and severity were assessed on basis of the MINI Diagnostic Interview. DUDIT, Drug Use Disorders Identification Test; SUD, substance use disorder; SD, standard deviation; f, female; m, male.*

### Confirmatory Factor Analysis

Across all participants, the two-factor model had the highest CFI and was the only one of the three models in which all model fit indices were considered good or acceptable (CFI = 0.995, SRMR = 0.055, RMSEA = 0.059, WRMR = 0.603). The model fit indices for all three models are displayed in Table 2. Furthermore, the two-factor model showed an acceptable to good fit when assessed separately for SUD patients and non-SUD patients as well. See Supplementary Material for details. Based on these results the SWLS factor was added to the two-factor model, which led to the discriminant validity model containing three factors (factor1 = DUDIT-C, factor 2 = DUDIT items 5–11, and factor 3 = SWLS items 1–5). Across the whole sample, the discriminant validity model showed good fit in all indices (CFI = 1.00, RMSEA = 0.000, WRMR = 0.742) except the SRMS, which was acceptable (SRMR = 0.074). The acceptable to good fit of the discriminant validity model was also present when applied to the SUD patient subsample and the non-SUD patient subsample, see Supplementary Material.

**TABLE 2:** Results of the confirmatory factory analysis with three different models, across *n* = 124 participants.

Model	Chi-square/df ratio	CFI	SRMR	WRMR	RMSEA (90% CI)
1-factor	3.18	0.976**	0.90*	1.058	0.133 (0.108–0.158)
2-factor	1.43**	0.995**	0.055*	0.603**	0.059* (0.017–0.090)
2-factor plus total score	7.15	0.932*	0.147	1.837	0.224 (0.201–0.247)

The symbol ** indicates good model fit according to Schermelleh-Engel et al. (2003) and DiStefano et al. (2018).

The symbol * indicates acceptable model fit according to Schermelleh-Engel et al. (2003) and DiStefano et al. (2018).

*df, degrees of freedom; CFI, comparative fit index; SRMR, standardized root mean squared error; WRMR, weighted root mean squared error; RMSEA, root mean square error of approximation.*

### Area Under the Curve

**FIGURE 1:**
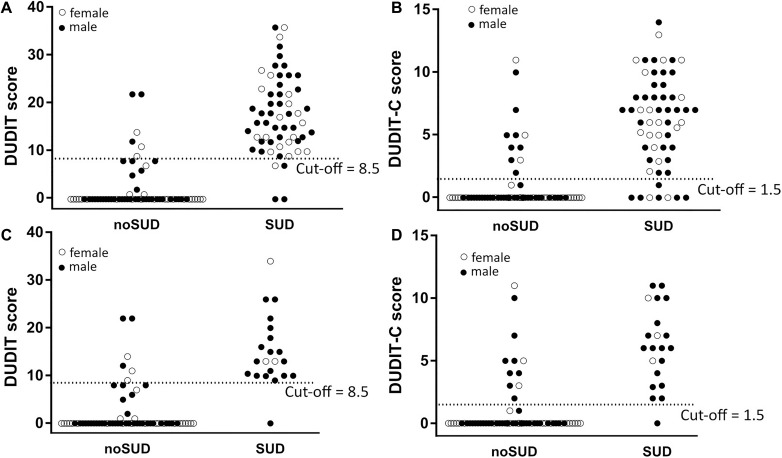
Distribution of raw values of the DUDIT and DUDIT C. Single dots represent the results from a single participant. A dotted line marks the optimal cut-off score based on Youden’s Index *J*. **(A)** Raw values and cut-off for DUDT in total sample. **(B)** Raw values and cut-off for DUDIT-C in total sample. **(C)** Raw values and cut-off for DUDIT in mild and moderate cases. **(D)** Raw values and cut-off for DUDIT-C in mild and moderate cases.

**FIGURE 2:**
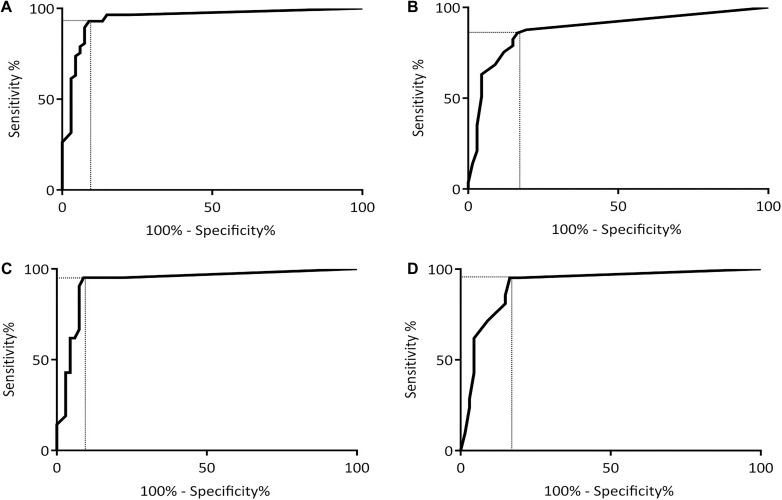
ROC curves for the DUDIT and the DUDIT-C with sensitivity plotted against 100%-specificity. Dotted lines mark levels of sensitivity and 100%-specificity that are in line with optimal cut-off score based on Youden’s Index *J.* **(A)** ROC curve for the DUDIT in the complete sample. **(B)** ROC curve for the DUDIT in the sample of mild and moderate cases. **(C)** ROC curve for the DUDIT-C in the complete sample. **(D)** ROC curve for the DUDIT-C in the sample of mild and moderate cases.

The DUDIT-total and DUDIT-C raw values distributions are shown in Figure 1. The DUDIT-total and DUDIT-C ROC curves including the AUC can be found in Figure 2.

For the DUDIT-total the AUC was larger than 0.9 with AUC = 0.95, 95% CI (0.90, 0.99), while for the DUDIT-C the AUC was slightly lower with AUC = 0.88, 95% CI (0.81, 0.95). Compared to the whole sample the AUC for the DUDIT-total is slightly smaller when comparing only patients with mild and moderate SUD to participants without a SUD: AUC = 0.93, 95% CI (0.86, 1.0). However, the DUDIT-C shows a larger AUC when only including the mild and moderate SUD cases: AUC = 0.91, 95% CI (0.84–0.98).

### Cut-off Values

Based on Youden’s Index *J* optimal cut-off values were calculated for the DUDIT-total and DUDIT-C. For the DUDIT-total the optimal cut-off was at a value of 8.5 (sensitivity = 0.93, specificity = 0.91, *J* = 0.84), meaning 93% of SUD patients were correctly classified as SUD patients, while 9% of non-SUD patients were falsely classified as SUD patients. For the DUDIT-C the optimal cut-off was at a value of 1.5 (sensitivity = 0.86, specificity = 0.84, *J* = 0.70), with which 86% of SUD patients were correctly classified and 16% of non-SUD patients incorrectly classified. In mild or moderate cases our analysis resulted in the same cut-off values as were determined for the complete sample: Based on Youden’s Index *J* the optimal cut-off value for distinguishing patients with a mild or moderate SUD from patients without a SUD is a DUDIT-total score of 8.5 (sensitivity = 0.95, specificity = 0.91, *J* = 0.86). Additionally, the optimal cut-off to diagnose patients with a mild or moderate SUD with the DUDIT-C was at a value of 1.5 (sensitivity = 0.95, specificity = 0.84, *J* = 0.79).

## Discussion

This study showed, that the DUDIT can be reasonably separated into two factors consisting of the first four items related to consumption (DUDIT-C) and items 5–11 related to drug-related problems. Furthermore, we showed that the complete DUDIT as well as the DUDIT-C have outstanding diagnostic accuracy for detecting SUDs regardless of severity, in German adolescent psychiatry patients based on DSM-5 criteria. Additionally, the DUDIT shows excellent diagnostic accuracy for detecting patients with mild or moderate SUDs. Finally, we determined a DUDIT cut-off value across all participants of 9, meaning any patient with a DUDIT score higher than 8 is likely to fulfill the diagnostic criteria for a SUD. For the DUDIT-C, our results indicate an optimal cut-off value at a score of 2. If only differentiating between patients without a SUD and patient with mild or moderate SUD the same cut-off value (9 for the DUDIT and 2 for the DUDIT-C) can be used.

Overall, the DUDIT and DUDIT-C are instruments with excellent discriminative power, regarding adolescent psychiatric patients, and adolescent SUD patients, making them suitable for clinical practice. However, our cut-off values are based on Youden’s Index, which aims for a balance between sensitivity (the ability to correctly identify SUD patients) and specificity (the ability to correctly exclude non-SUD patients). Yet, in adult SUD patients DUDIT cut-off values (based on DSM-IV), often have a higher sensitivity than specificity (see Hildebrand, 2015 for an overview), which might reflect a desire to focus on the ability to correctly identify SUD patients. On the other hand, in a public health setting it might be more important to focus on specificity, excluding non-SUD patients correctly, to establish accurate estimates of prevalence. This focus on specificity might be relevant when trying to investigate local patterns of SUD distribution or when aiming to offer selected SUD-specific treatment options.

Our CFA supported the division of the DUDIT into two factors and indicated good discriminant validity of the DUDIT compared to the SWLS. This structure has previously been shown for the similar questionnaire for alcohol use disorders, the AUDIT (Liskola et al., 2018). Previous research regarding the factor structure of the DUDIT, mainly supported a one-factor models, over two- or three-factor models (Evren et al., 2014a,b; Hildebrand and Noteborn, 2015), which could be the result of different factor composition across studies. No previous studies assessed the specific two-factor model supported by our results. One reason for this disparity might be specific sample characteristics. Our sample included a large subset of patients with a severe SUD and only few patients with mild or moderate SUD. This distinction might be reflected in our two-factor model in the sense, that one factor (DUDIT-C) might respond to the patients with mild or moderate SUD while the second factor (DUDIT items 5–11) might respond to the patients with a severe SUD. Additionally, the samples in previous studies were compromised entirely (Evren et al., 2014a,b) or to a very large extend (Hildebrand and Noteborn, 2015) of male participants, while our sample included a more balanced split in male and female participants. The homogeneity of the previously investigated samples might have contributed to the support of one-factor models.

This paper includes the first psychometric assessment of the DUDIT consumption questions (DUDIT-C). Although the DUDIT-C has been used as a screening tool for substance use in several studies with adults (Sinadinovic et al., 2012, 2014a; Gidhagen et al., 2017; Bright et al., 2018) and adolescents (Hillege et al., 2010; Willem et al., 2011) no psychometric assessment, apart from internal consistency, in adolescents (Hillege et al., 2010) has been published. Based on our results the DUDIT-C has high diagnostic accuracy in adolescents, but displays reduced sensitivity and specificity compared to the complete DUDIT. The DUDIT-C can therefore be considered a valuable screening tool in time-sensitive settings. Furthermore, this study is the first published psychometric assessment of the German version of the DUDIT. While previous studies in German adults (Schäfer et al., 2017; Spencer et al., 2018; Dyba et al., 2019) and German adolescents (Basedow et al., 2020) have used the DUDIT as a measure or screening tool, none have reported on the psychometric properties. Additionally, previous research has assessed the DUDIT on basis of the DSM-IV criteria for substance abuse and dependence (see Hildebrand, 2015 for an overview), which makes this study the first psychometric assessment using the updated DSM-5 criteria with three levels of SUD.

In addition to assessing the diagnostic accuracy for the whole sample, we investigated DUDIT and DUDIT-C performance in a subsample consisting of patients with mild or moderate SUD. In these patients, the DUDIT cut-off value was related to a higher sensitivity but the same level of specificity, meaning that the DUDIT was more likely to correctly identify patients with a mild or moderate SUD, and had the same likelihood of incorrectly classifying non-SUD patients as having a mild or moderate SUD. Similarly, the DUDIT-C also showed a higher sensitivity and the same level of specificity in cased with mild or moderate SUD. Additionally, the DUDIT-C showed higher diagnostic accuracy in these mild or moderate patients than in the complete sample. These results might be an indication, that the DUDIT-C is particularly valuable for settings where adolescents with less severe forms of SUD are seen, e.g., a general practitioner, or youth counselor office.

Comparing our cut-off, sensitivity, and specificity values to previous research is of limited use, since previous studies did not use DSM-5 diagnostic criteria as a comparison (Hildebrand, 2015). Nonetheless, our cut-off values for any DSM-5 SUD are slightly lower than values for DSM-IV dependence (Durbeej et al., 2010) or any DSM-IV drug use disorder (Evren et al., 2014a,b). This difference is likely due to these three studies sampling participants from criminal justice settings instead of a psychiatric care environment as we did. Unfortunately, no other cut-off values for adolescents have been established either for the DUDIT or DUDIT-C, which highlights the need for additional research into substance use specific instruments for adolescents.

The high diagnostic accuracy we determined for the DUDIT is in line with the accuracy for other screening instruments for adolescents like the Brief Screener for Tobacco, Alcohol, and other Drugs (BSTAD), the Alcohol, Smoking, and Substance Involvement Screening Test (ASSIST), or the CRAFFT (Knight et al., 2002; Kelly et al., 2014; Gryczynski et al., 2015). All of these questionnaires, as well as the DUDIT are freely available in various translations. The main difference between the DUDIT and the other screening instruments is the DUDITs’ focus on illegal drugs. While the other screening instruments include questions on the use of alcohol and tobacco, the DUDIT explicitly asks only for answers related to the use of illegal drugs. This structure also is responsible for the major weakness of the DUDIT, namely the fact that the DUDIT does not specify which specific drug the questions are related to. Consequently, in scoring the DUDIT one can only make judgments about drug use problems without specifying the drug. At the same time, this structure makes the DUDIT very fast in its administration which alleviates one of the main barriers to accurate SUD screening, time constraints (Palmer et al., 2019). This time constraint is reduced even further with the use of the DUDIT-C, which only comprises four questions. Actually, an extended version of the DUDIT has been developed which includes questions about the type of substances used and the frequency of use (DUDIT-E) (Berman et al., 2007). The inclusion of all three instruments could constitute a diagnostic three-step procedure: use the DUDIT-C for a quick first screening. If the cut-off is reached, apply the complete DUDIT, and if this cut-off is reached as well, use the DUDIT-E for an extended assessment.

### Limitations and Future Research

First, we focused on a very specific sample, namely psychiatric patients. This subpopulation is pre-selected in so far, as they showed some disordered behavior in the past that made them or their parents seek psychiatric care for their disorder. Therefore, our study fails to include a more general population of adolescents who might fulfill SUD criteria but are not affected enough to seek treatment.

Second, our focus was on screening for any level of severity of a SUD, thus any health care professional who wants to screen for a specific level of severity of SUD could not use the values we calculated. To screen for specific levels of severity, new cut-off values need to be determined. This issue also relates to the first limitation, since it highlights the need to establish cut-off values for each level of SUD severity separately.

Third, in our sample only few patients presented with a mild or moderate SUD, which means our results regarding the cut-off values in non-severe SUD patients should be considered preliminary. Since the sample size in that group was small, future research should take care to repeat a similar analysis with adolescent patients presenting only with a mild or moderate SUD.

Fourth, a considerable proportion of SUD patients were not classified as such by the DUDIT (7%) or the DUDIT-C (14%). While unfortunate, this is an expected proportion of failure that has been shown to occur at similar rates in the AUDIT (Kuitunen-Paul et al., 2018). This non-diagnosis might be a result of a social desirability or recall bias, which are known to skew self-report data (Althubaiti, 2016).

Finally, while the majority our patients reported understanding the DUDIT well, *n* = 11 (14%) participants reported that they had problems understanding the DUDIT items and instructions (a score below 5 on a 10-point scale). It is possible that these participants misunderstood the questionnaire and did not answer it correctly. For example, they might have answered the questions while thinking of their use of legally available drugs like alcohol or nicotine.

## Conclusion

This study is the first evaluation of the DUDIT and DUDIT-C in a German sample as well as a sample of adolescent psychiatric patients, based on DSM-5 criteria. We found that the DUDIT and DUDIT-C are easily accessible, free-to-use, screening instruments for SUDs that have high diagnostic accuracy in a German adolescent, psychiatric population.

## Data Availability Statement

The raw data supporting the conclusions of this article will be made available by the authors, without undue reservation.

## Ethics Statement

The studies involving human participants were reviewed and approved by the Institutional Review Board of the University Hospital C. G. Carus Dresden (EK 66022018). Written informed consent to participate in this study was provided by the participants’ legal guardian/next of kin.

## Author Contributions

LB analyzed the data and wrote the manuscript. SK-P participated in writing the manuscript, data analysis, and contributed to the discussion. AE participated in writing the manuscript, preparation of figures, and contributed to discussion. VR participated in writing the manuscript and contributed to discussion. YG designed the study, participated in writing the manuscript, and contributed to discussion. All authors contributed to the article and approved the submitted version.

## Conflict of Interest

During the past 36 months and unrelated to the presented analyses and data, SK-P received author fees (Mabuse Verlag). The remaining authors declare that the research was conducted in the absence of any commercial or financial relationships that could be construed as a potential conflict of interest.
