# Supplementary material for: Diagnostic Accuracy of the Drug Use Disorder Identification Test and Its Short Form, the DUDIT-C, in German Adolescent Psychiatric Patients
Source: Front Psychol. 2021 Jun 4;12:678819. doi: 10.3389/fpsyg.2021.678819 (PMC8212997; doi:10.3389/fpsyg.2021.678819)
Supplement: Supplementary file 1 [file Table_1.docx]

Supplementary Material

Supplementary Table 1. Results of the confirmatory factory analysis with three different models.

| **Model** | **Chi-Square (df)** | **p-value** | **CFI** | **SRMR** | **WRMR** | **RMSEA [90% CI]** |
| --- | --- | --- | --- | --- | --- | --- |
| *SUD patients (n = 57)* |  |  |  |  |  |  |
| **1-factor** | 159.776 (44) | <.001 | .710 | 0.174 | 1.242 | 0.217 [0.181 – 0.253] |
| **2-factor** | 62.037** (43) | .030* | .952* | 0.114 | 0.696** | 0.089* [0.029 – 0.135] |
| **Complex model** | 64.568** (44) | .023* | .948* | 0.119 | 0.750** | 0.091* [0.035 – 0.137] |
| **Discriminant validity model (n = 56)** | 89.706** (87) | .400** | .994** | 0.106* | 0.815** | 0.024** [0.000 – 0.078] |
| *Non-SUD patients (n = 67)* |  |  |  |  |  |  |
| **1-factor** | 84.750** (44) | <.001 | .999** | 0.493 | 1.219 | 0.118 [0.080 – 0.156] |
| **2-factor** | 70.295** (43) | .005* | .999** | 0.440 | 1.026 | 0.098* [0.054 – 0.138] |
| **Complex model** | 124.826 (44) | <.001 | .997** | 0.396 | 1.616 | 0.167 [0.133 – 0.202] |
| **Discriminant validity model (n = 52)** | 98.520** (87) | .405** | .986** | 0.216 | 0.814** | 0.024** [0.000 – 0.081] |

*** indicates good model fit according to (Schermelleh-Engel et al., 2003; DiStefano et al., 2018); * indicates acceptable model fit according to (Schermelleh-Engel et al., 2003; DiStefano et al., 2018); CFI, comparative fit index; SRMR, standardized root mean squared error; WRMR, weigthed root mean squared error; RMSEA, root mean square error of approximation.*
